# Supplementary material for: Chromothripsis during telomere crisis is independent of NHEJ, and consistent with a replicative origin
Source: Genome Res. 2019 May;29(5):737–49. doi: 10.1101/gr.240705.118 (PMC6499312; doi:10.1101/gr.240705.118)
Supplement: Supplemental Material [file supp_gr.240705.118_Supplemental_file_1.zip › contigs/annotated_contigs/DB51/contig.2.DB51_length_394_mean_cov_7.99492385787.docx]

**DB51_length_394_mean_cov_7.99492385787**

```
TTTTTCTGGAGCCAGAATGCAACTCCAACTAAAGCCCTCTGCTCCATTTCTCTTAAGTGCAGACCCATCCTTAATCCAGTGGGCACAAT
 >chr4:133278353-133278615 - E=2e-146
CTAATCAGCTGCCAGCAAATATAAAGCAGGCAGAAAAACATGAAAAGGTGACAGTGGCCTAGCCTCCCAGCCTACATCTTTCTCCCATG

CTGGATGTTTCCTGCCCTCAAACATCAGACTCCAAGTTCTTCAGTTTTGAGACTTGGACTGACTCTCCTTGCTCCTAGGGCT|TG|ACC
                                                                                   >chr
CATGCTGCCATGAGCCTTTCCACCTTTGTCAGAGGAGATAAATACCTCATTGCCTGAGGCTACAATGATGGTCTCCCCTGAGGCAGGGG
4:133277432-133277566 - E=3e-69
AGACTTTTGACAGGCAAAGTAATATTGATTATCCTCAGGA
```
